# Machine learning applications in radiation oncology: Current use and needs to support clinical implementation

**DOI:** 10.1016/j.phro.2020.11.002

**Published:** 2020-11-30

**Authors:** Charlotte L. Brouwer, Anna M. Dinkla, Liesbeth Vandewinckele, Wouter Crijns, Michaël Claessens, Dirk Verellen, Wouter van Elmpt

**Affiliations:** a University of Groningen, University Medical Center Groningen, Department of Radiation Oncology, Groningen, The Netherlands; b Department of Radiation Oncology, Amsterdam University Medical Center, VU University, The Netherlands; c Department Oncology, Laboratory of Experimental Radiotherapy, KU Leuven, Leuven, Belgium; d Radiation Oncology, UZ Leuven, Leuven, Belgium; e Iridium Network, Wilrijk (Antwerp), Belgium; f Faculty of Medicine and Health Sciences, University of Antwerp, Antwerp, Belgium; g Department of Radiation Oncology (MAASTRO), GROW School for Oncology, Maastricht University Medical Centre+, Maastricht, The Netherlands

**Keywords:** Artificial intelligence, Machine learning, Radiotherapy, Survey, Commissioning, Quality assurance, Clinical implementation

## Abstract

**Background and purpose:**

The use of artificial intelligence (AI)/ machine learning (ML) applications in radiation oncology is emerging, however no clear guidelines on commissioning of ML-based applications exist. The purpose of this study was therefore to investigate the current use and needs to support implementation of ML-based applications in routine clinical practice.

**Materials and methods:**

A survey was conducted among medical physicists in radiation oncology, consisting of four parts: clinical applications (1), model training, acceptance and commissioning (2), quality assurance (QA) in clinical practice and General Data Protection Regulation (GDPR) (3), and need for education and guidelines (4). Survey answers of medical physicists of the same radiation oncology centre were treated as a separate unique responder in case reporting on different AI applications.

**Results:**

In total, 213 medical physicists from 202 radiation oncology centres were included in the analysis. Sixty-nine percent (1 4 7) was using (37%) or preparing (32%) to use ML in clinic, mostly for contouring and treatment planning. In 86%, human observers were still involved in daily clinical use for quality check of the output of the ML algorithm. Knowledge on ethics, legislation and data sharing was limited and scattered among responders. Besides the need for (implementation) guidelines, training of medical physicists and larger databases containing multicentre data was found to be the top priority to accommodate the further introduction of ML in clinical practice.

**Conclusion:**

The results of this survey indicated the need for education and guidelines on the implementation and quality assurance of ML-based applications to benefit clinical introduction.

## 1 Introduction

Machine learning (ML) applications are evolving from the academic research departments and turning into commercially available products. For radiotherapy, several (commercial) solutions are currently offered for various applications such as automatic segmentation of organs-at-risk, treatment planning and generation of synthetic images [1], [2], [3].

Medical physicists have a long tradition in implementing (novel) technology in clinical routine practice, supported by established Quality Assurance (QA) programs [4], [5]. Currently, many clinics are starting to use ML applications and are faced by the various steps needed for implementation in clinical practice. Traditionally, technology was mainly based on deterministic and atlas-based models, whereas with artificial intelligence (AI) this predictable nature might not always be guaranteed. Mathematical education of AI techniques is not in the standard curriculum of medical physicists and therefore some medical physicists may feel uncomfortable with this new technology [6]. Another difference is that for some ML models such as those used for automatic segmentation or treatment planning are usually trained on large (clinical) datasets. This might be unfamiliar territory for the physicist and requires knowledge on the current General Data Protection Regulation (GDPR) and privacy legislation [7]. Furthermore, current Medical Device Regulation (MDR) [8] will imply additional regulations and stress the need for (prospective) risk analysis methods such as a (health) failure mode and effect analysis ((H)FMEA) as described by AAPM Task Group 100 [9].

In this paper we aim to reveal the current needs for medical physicists and present the gap in knowledge that needs to be bridged for successful introduction of ML-based applications in clinical practice.

## 2 Materials and methods

A survey was designed to determine to what extent ML-based applications are currently implemented in clinical practice, how medical physicists see their role in the near future and what is needed for safe and efficient clinical introduction. The survey was centred around (1) key applications relevant to radiation oncology: contouring, treatment planning, machine quality assurance (QA), synthetic CT generation, or other; (2) model training, acceptance and commissioning, and risk analysis; (3) Model monitoring (QA) in clinical practice and GDPR [7]; and (4) need for education and guidelines. If responders did not use AI in clinic yet, they were asked if they started preparing the use of AI in clinic. Questions in part 2, 3 and 4 were asked to current AI users and responders preparing for the use of AI in clinic.

The final web-based questionnaire (Survey in Supplemental Material 1) was distributed and promoted via ESTRO physics mailing list (containing 2774 physicists in total) and was open from January until April 2020 to all medical physicists (mainly in Europe but input from all was taken).

In the invitation besides users of AI technology, also responders that did not yet apply ML/AI in clinical practice but were working towards clinical application were asked to complete the survey as how they foresee its application in clinical practice.

Responders were included in the analysis when a complete, unique response was provided or only some isolated questions had not been answered. Multiple responders of the same radiation oncology centre were merged in case of duplicate responses per application, but were treated as a separate unique responder in case reporting on different AI applications.

## 3 Results

**Fig. 1:**
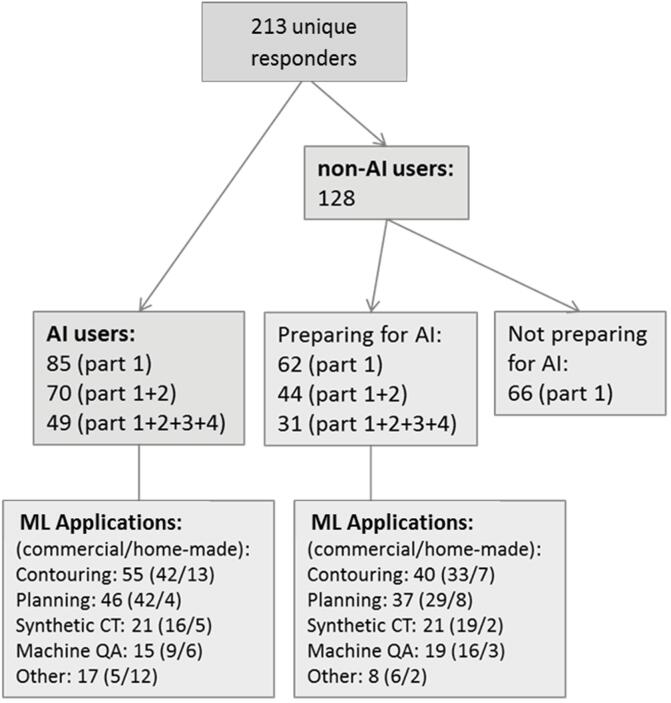
Number of responders to the different parts of the survey (see Supplemental Material 1), and the number of specific applications indicating the ratio between commercial and home-made applications. AI: Artificial Intelligence, ML: Machine Learning, QA: Quality Assurance.

In total, 257 medical physicists (‘responders’) from 246 different radiotherapy departments over 40 different countries responded to the survey (Table S1). Forty-four responders were excluded from the analysis because of missing data in part 1 of the questionnaire, leading to 213 unique responders from 202 radiotherapy departments (Fig. 1).

Only 37% (85) of the responders indicated that AI software was used in their clinic (Fig. 1), with 15% (34) for at least two applications, 8% (18) for at least three and 3% (7) for more than three applications.

The main reason for clinics which were not preparing on the use of AI software was the lack of information/knowledge on how to do this, no software options or lack of resources or time (35/66). Six responders mentioned resistance or scepticism against AI among staff and sixteen responders did not specify a reason. Still, 32% (21/66) of this non-user group expected to have AI in the clinic within the next two years.

The main reason to introduce AI software was time saving and quality improvement (both 26 times), followed by increased consistency (18) and saving resources (8).

The largest application of AI in clinic was contouring (Fig. 1), mainly using commercially available software. Automation of treatment planning using AI was second ranked, followed by AI for synthetic CT generation and machine QA. In the AI user group, a minority of 26% of the applications was home-made, while in the prepare for AI group this was even less (18%).

For contouring and synthetic CT purposes, deep learning techniques were the most popular techniques while for planning and machine QA, one mostly used other machine learning techniques. It is also noteworthy that 22% did not know which specific ML technique was used.

Apart from the four main categories, 16/25 responders provided the purpose of the application for ‘other’ purposes. Responders reported on mainly in-house build applications regarding clinical decision support (5), patient specific QA (4), (MR) image analysis/reconstruction (4), plan adaptation (2) and education (1).

### 3.1 Model training, acceptance & commissioning and risk analysis

For part 2 of the survey containing questions on acceptance and commissioning of AI tools, a total of 114/147 responders that were using or preparing to use AI tools answered the related questions (See Supplement, Questions: 9, 10 and 11).

**Fig. 2:**
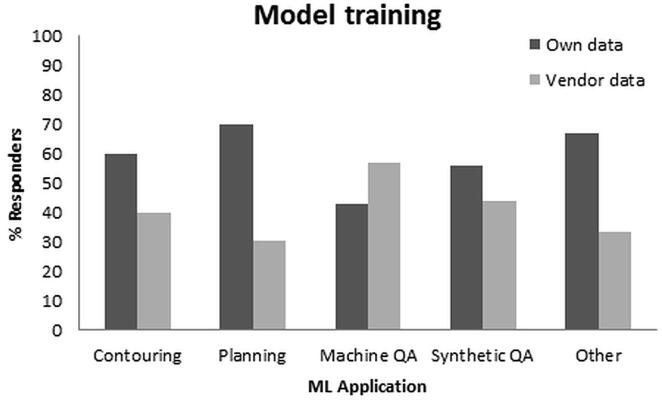
Involvement of responders in model training per machine learning (ML) application, i.e. using their own data or vendor data.

Model training was performed mostly by using the institute’s own data (Fig. 2). For all applications, the number of data points in model training was most often < 100 patients. In 10% of cases, more than 200 patients were used.

The majority of responders did verify upfront the input data for the model training, mainly by visual inspection and manual correction of the data. Three responders mentioned an outlier analysis by reviewing statistics, and three others excluded irregular patients/setup positions from the training set.

Commissioning and evaluation of the output of the model was typically done by subjective evaluation of the output data (51%) (e.g. for contouring and treatment planning the results were inspected by a radiotherapy technologist (RTT), medical physicist or radiation oncologists). Time saving analysis was done in 35% of cases and objective measures in 32%. In 18% of cases, all three have been done, and 10% did not respond to this question (indicating none of the three options was done). Only eight responders (7%) specified the number of patients (2x10-20, 2x20-50, 4x greater than 50 patients) and the type of objective measures (Table 1).

**Table 1:** Objective measures for acceptance and commissioning of machine learning applications for contouring and planning.

	#Responders
Contouring	
Dice similarity coefficient	6
Hausdorff Distance	5
Surface Dice/ added path length/ false positives/negatives	3
COM shift	1
Planning	
DVH comparison	2
Plan quality measures	2

COM: center of mass, DVH: dose volume histogram.

The minority (22%) of responders performed a (prospective) risk analysis method prior to clinical introduction of the AI. The risk analysis methods reported were listed in Table 2, the most used method was a (H)FMEA .

**Table 2:** Risk analysis for machine learning applications.

	#Responders
(H)FMEA	5
Vendor performed risk analysis	2
Embedded in QA program	2
QMS	1
OUH NHS risk assessment	1
Risk and hazard analysis	1
Risk assessment workflow	1
General risk assessment physics/IT	1

(H)FMEA: Healthcare Failure Mode and Effect Analysis, QA: Quality Assurance, QMS: Quality Management System, OUH NHS: Oxford University Hospitals National Health Service, IT: Information Technology.

### 3.2 Model monitoring in clinical practice (case specific QA/ routine QA)

The last part of the questionnaire (3 + 4) (See Supplement, Question 12 and further) was completed by in total 80 responders (allowing for a maximum of 1 missing answer).

Thirty-nine percent of responders indicated no regular QA on the AI application, while 41% reported to monitor or evaluate manual interactions performed. Only 15% indicated to perform regular QA, although the majority indicated the type of tests are in development, this is ‘work in progress’, or qualifications such as ‘need to build trust over the years’. Two physicists responded to synchronize the AI-based verification workflow with classical (i.e. non-AI) workflows by embedding dry runs and manual interventions in the process. One centre reported on a weekly joint multidisciplinary meeting including machine learning experts to discuss all aspects of models and post implementation data gathering and analysis. The detailed responses describing QA in clinical practice were provided in Table 3. For planning, one responder reported on a manual checklist to verify the dose volume histogram (DVH), couch positioning, normalization, beam angles, field sizes etc. Medical dosimetrists start this check procedure and physicists perform a final validation.

**Table 3:** Model monitoring Quality Assurance (QA) measures during clinical use of machine learning applications.

	#Responders
Assessment of edits/modifications	4
Model constancy testing/ end-to-end performance	2
AI workflow as parallel process for offline assessment	2
Time saving analysis for first clinical patients	1
Home-made application for QA of upgrades (contouring)	1
Checksum checks (contouring and planning)	1
Six-month check with a shadow database (synthetic CT)	1

In the large majority (86%) of the cases, human observers were still involved in daily clinical use for quality check of the output of the AI algorithm.

### 3.3 GDPR and patient privacy

Only 38% of responders considered how to deal with GDPR related issues concerning (in-house) applications. Four responders indicated data was anonymized before model training and training was carried out within the institution. Three responders indicated to have institutional review board (IRB) approval process in place and three others to have consulted regional/ national ethics committees.

Also sharing data with 3rd parties (e.g. other institutes or vendors) was scored differently, the majority of responders (43%) indicated this was possible but only with informed consent of the patient and/or when a data sharing agreement was in place. Twenty-three percent indicated sharing data was not possible for them at all.

Some responders pointed out the radiotherapy community should ultimately concentrate the learning and knowledge on as few systems as possible, help (collaborations for) data sharing and then aid the deployment of this technology to the wider community.

### 3.4 Need for education and guidelines

**Fig. 3:**
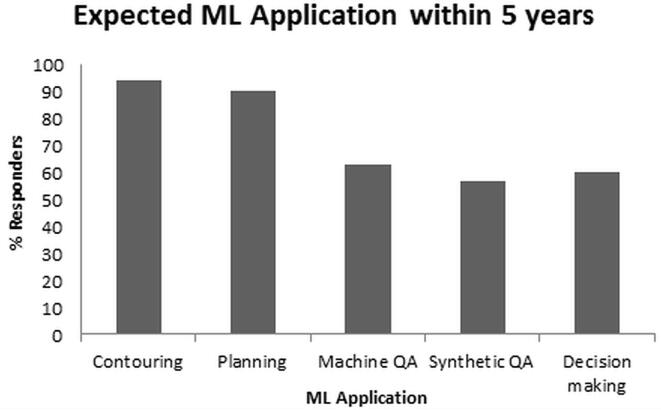
Percentage of responders expecting the specific machine learning (ML) applications in responders’ clinic within the next 5 years.

A lack of knowledge about machine learning became clear in the question ‘How would you describe your level of expertise on machine learning/deep learning?’. Forty-one percent answered this question with ‘Beginner’ and 31% answered with ‘Basic’. At the same time, ML-based applications are rapidly entering the clinic (Fig. 3).

For a broader adoption of AI in clinic, 40% agreed that ‘Training of medical physicists’ is the most important need, followed by ‘Larger databases including multicenter data’ (23%). ‘Employ AI expert’ scored average and was selected by the larger centers, whilst others indicated the medical physicist has the potential to become the AI expert in the hospital setting.

The majority of responders indicated that ‘a course for physicists to aid in commissioning and implementation of models in the clinic’ (76%), ‘a multidisciplinary course on how to use models in the clinic and what to expect from it’(67%), and ‘a course for physicists to increase knowledge or get started on building and training your own models’ (59%) would be useful. Individual comments indicated such a course should encompass ethics/legislation and guidelines for using AI in hospital setting. Other suggestions were help with sharing of data and models.

Eighty-seven percent agreed that professional organisations such as ESTRO should help to provide guidelines on QA and the use of machine learning/deep learning. Although some responders remarked that it might be too early for a full set of comprehensive guidelines but a first start would be helpful to elaborate the discussion on this topic.

## 4 Discussion

This study provided insight into the current use and needs to support clinical implementation of ML-based applications in radiation oncology by incorporating 213 survey responders who were physicists from 40 different countries.

Thirty-seven percent was using ML in clinical practice, mainly for contouring and treatment planning, with the main motivation to save time and improve quality. One of the most frequent mentioned reasons for not (yet) introducing ML in clinic was lack of knowledge on the commissioning process. AI technology is rapidly entering the radiotherapy field and not everybody is yet involved at the same expertise or implementation level.

The response rate to this survey was relatively low (~10%) if one only considers the size of the ESTRO mailing list. However it included 202 different radiation oncology departments which reflects 23% of the total number of radiation oncology departments in Europe (8 7 2)[10].

Out of the 213 unique responders, only 80 responders were able to answer the full questionnaire. Open text fields for further specification of risk analysis, acceptance, commissioning and QA were filled by < 20 responders, which is a limitation of this survey report. Still, it provides useful suggestions of early adopters and will be a good starting point for further discussion within the community.

The majority of responders planned to introduce ML-based contouring and planning in the short term (e.g. 2–5 years) into their clinic. This is most likely due to the large expectations in terms of workflow improvement, but also because the end-result (i.e. an organ segmentation or a treatment plan) is something that can be verified or benchmarked against the conventional/ standard workflow.

For model training, implementation and commissioning, it is still an open field how to best introduce AI based technology in clinical routine. Many physicists follow their commissioning guidelines as they would for other technology introductions. However, one needs to be cautious because typically large amount of (patient) data is needed for this and manual curation is labour intensive. Especially, given the fact that medical physicists are often not familiar with the behaviour of AI models, a subjective evaluation of the outcome data could increase the implementation time if multiple scenarios need to be evaluated.

Risk analysis and QA in clinical practice are an essential component of novel technology introduction in health care. Although the current Medical Device Regulations (MDR) stress the need for proper (prospective) risk analysis methods, only 22% of responders performed a risk analysis. Consensus between and guidance to responsible physicists on this topic seems to be required, with special attention to ML-based applications.

QA in clinical practice depends heavily on the method implemented but also on the ‘trust’ that is present in clinics. Institutes that are at the forefront of development of technology in house typically know the limitations better of the methodology compared to centres that are implementing commercially available tools that are not in-house trained. Guidelines to support the medical physicist in the introduction of AI into clinical practice should therefore aim to include the steps needed for all scenarios (home-made, vendor collaboration or commercial model).

Considering GDPR and patient privacy [7], this is a concern that many have not yet found a way to efficiently integrate. Many centres do have data-sharing agreements in place to be able to share patient data with vendors or other institutes, but also 23% indicate that they are not allowed by their hospital to share data. This latter might make tuning and optimization of AI technology cumbersome or sub-optimal. Data sharing is therefore a remaining challenge to be addressed in order to create and curate large datasets, as was also underlined by previous work by Feng et al. [11]. The authors described opportunities, requirements and needs regarding machine learning in radiation oncology, and concluded that updating our curriculums regarding ML algorithms will be increasingly important. The review by He et al. [12] on key practical issues surrounding the implementation of AI into existing clinical workflows anticipates that the GDPR may potentially slow down AI implementation in healthcare in the short term owing to the stricter regulatory standards, but it may facilitate implementation over the long term by promoting public trust and patient engagement.

As a result from this survey, education and guidelines were revealed to be highly needed for a broader adoption of ML-based applications in the clinic. Professional organisations (e.g. ESTRO, AAPM, EFOMP) play a role in this field to raise the educational level of medical physicists. ESTRO and AAPM working groups defined ‘adoption of artificial intelligence and automation’ as one of the grand challenges of medical physics in radiation oncology [13] and underlined the need for basic knowledge of AI in the curricula for medical physicists [14]. The EFOMP working group on AI aims to ensure that medical physicists’ professional education, continuous training and competence will follow this significant global development [15].

With the first commercial radiotherapy products already being used in clinical practice, the need for guidelines on commissioning and implementation is urgent. Recent recommendations for implementation and quality assurance of AI-based applications aim to support clinical teams during implementation of machine learning models in the radiotherapy workflow for contouring, planning and synthetic CT [16].

Many responders indicated the need for education of the physician as well (67% thinks professional organizations should organize a ‘multidisciplinary course’). Training of physicians and RTTs was seen as the third priority after training of medical physicists and facilitating multi-center databases. Chamunyonga et al. proposed a number of aspects to ML approaches that could be embedded in clinical education programs [17]. Healthcare providers will need to understand the algorithms, datasets underlying their outputs and limitations of the application, to maximize their functioning on human–machine teams [12].

In conclusion, the results of this survey underlined three important aspects; Firstly, AI has become part of the radiotherapy clinic for 37% of the responders and is expected to expand rapidly within the next five years, secondly, there is no clear consensus on specific methods for commissioning and QA, and thirdly, medical physicists indicate the need for guidance regarding commissioning and QA procedures for safe and efficient introduction of ML-based applications in clinical practice.

## Declaration of Competing Interest

The authors declare that they have no known competing financial interests or personal relationships that could have appeared to influence the work reported in this paper.
