# Supplementary material for: Machine learning applications in radiation oncology: Current use and needs to support clinical implementation
Source: Phys Imaging Radiat Oncol. 2020 Nov 30;16:144–8. doi: 10.1016/j.phro.2020.11.002 (PMC7807598; doi:10.1016/j.phro.2020.11.002)
Supplement: Supplementary data 1 [file mmc1.docx]

Supplemental material

**Supplemental Table 1**. Number of Radiation Oncology Departments using or preparing to use machine learning applications in clinical practice per country

| Radiation Oncology Departments and  Machine Learning Applications | | |
| --- | --- | --- |
|  | Clinical | Preparing |
| France | 8 |  |
| Italy | 7 | 5 |
| Netherlands | 8 | 3 |
| Spain | 7 | 2 |
| Australia | 5 | 4 |
| Belgium | 5 | 3 |
| United Kingdom | 4 | 3 |
| Denmark | 3 | 2 |
| Switzerland | 3 | 3 |
| United States of Amerika | 3 | 1 |
| Germany | 2 | 2 |
| Norway | 2 | 3 |
| Saudi Arabia | 2 |  |
| Austria | 1 |  |
| Azerbaijan | 1 |  |
| Canada | 1 | 1 |
| Finland | 1 |  |
| Greece | 1 | 1 |
| India | 1 | 2 |
| România | 1 |  |
| Russia | 1 |  |
| Indonesia | 1 |  |
| Sweden | 1 | 5 |
| Turkey | 1 |  |
| Ukraine | 1 |  |
| Bangladesh |  | 1 |
| Brasil |  | 1 |
| Chili |  | 1 |
| Ecuador |  | 1 |
| Hungary |  | 1 |
| Kenya |  | 1 |
| Malaysia |  | 1 |
| Morocco |  | 1 |
| Portugal |  | 1 |
| Singapore |  | 1 |
| South Korea |  | 1 |
| Sudan |  | 1 |
| Thailand |  | 1 |
| Anonymous | 8 | 4 |

Supplemental Material 1: Survey
