# Supplementary material for: Machine learning applications in radiation oncology: Current use and needs to support clinical implementation
Source: Phys Imaging Radiat Oncol. 2020 Nov 30;16:144–8. doi: 10.1016/j.phro.2020.11.002 (PMC7807598; doi:10.1016/j.phro.2020.11.002)
Supplement: Supplementary data 2 [file mmc2.pdf]

Part 1

Machine Learning/ Deep Learning in RT

Clinical Setting

1. What is your profession

2. Number of patients treated in your institution per year

3. Please fill in institution details

What is the name of your institution?

In which city is your institution located?

In which country is you institution located?

What is your e-mail address?

4. Is AI software in clinical use in your clinic?

- ○ Yes
- ○ No

If no AI software in clinical use:

5. Within which timeframe do you expect to use AI in your clinic

	<1y	1-2y	2-5y	don't know yet
Contouring	◯	◯	◯	◯
Planning	◯	◯	◯	◯
Machine qa	◯	◯	◯	◯
Synthetic CT	◯	◯	◯	◯
Other*	◯	◯	◯	◯

*please specify the application:

6. Are you preparing to use AI in clinic

◯ Yes (please fill out remaining of survey as how you plan/foresee the use of your AI application)

◯ No (please specify why not (yet))

If Yes AI software in clinical use <u>and</u> preparing to use AI in clinic:

5. What kind of AI software do you use in your clinic? please specify per application

	Commercial software	Home-made software
Contouring	◯	◯
Planning	◯	◯
Machine QA	◯	◯
Synthetic CT	◯	◯
Other*	◯	◯

6. What is the type of model (please specify most appropriate answer per application)?

	Machine Learning (non CNN)	Deep Learning (CNN)	I don't know
Contouring	◯	◯	◯
Planning	◯	◯	◯
Machine QA	◯	◯	◯
Synthetic CT	◯	◯	◯
Other*	◯	◯	◯

*please specify the application:

Part 2

7. Has your department been involved in model training?

	Yes, model is trained using our own data	No, model was purchased from vendor
Contouring	○	○
Planning	○	○
Machine QA	○	○
Synthetic CT	○	○
Other*	○	○

*please specify the application:

8. How many patients have been used for model training?

	<100	≥100	≥200	≥500	Vendor trained
Contouring	○	○	○	○	○
Planning	○	○	○	○	○
Machine QA	○	○	○	○	○
Synthetic CT	○	○	○	○	○
Other*	○	○	○	○	○

*please specify application:

9. Did you perform quality control of patients selected for model training?

	Yes*	No
Contouring	○	○
Planning	○	○
Machine QA	○	○
Synthetic CT	○	○
Other	○	○

*please specify what was done:

10. Has a risk analysis for your AI application been done?

- ○ No
- ○ Yes (please specify when and which type of risk assessment):

11. What kind of acceptance and commissioning tests did you perform (based on certain institutional/national/international guidelines or recommendations?) multiple options possible

- ☐ Scoring by RTT/ MD/ MP on acceptance/ quality
- ☐ Time saving analysis
- ☐ Objective measures

Please specify number of patients and which objective measures (if applicable):

Part 3

12. Do you perform regular QA on your AI-application?

☐ No

☐ Manual interactions are monitored/ evaluated

☐ Yes (please specify frequency and type of QA test):

13. Are human observers involved in daily clinical use of your AI application (quality check)?

○ Yes

○ No

14. Did you consider how to deal with GDPR for your (future) home-made applications?

○ Yes

○ No

○ I don't know

Please explain (Have you already taken measures?):

15. Privacy: Are you able to share your data with 3rd parties?

○ Yes

○ No

○ Only if informed consent is available

○ Only if informed consent and data sharing agreement is available

Optional comment:

16. What is your main reason to use AI in your clinic? Please rank in order of importance

- Save time
- Save resources
- Improve quality
- Increase consistency

Part 4

Machine Learning/ Deep Learning in RT

Need for training and guidelines

17. How would you describe your level of expertise on Machine Learning/ Deep Learning

- ○ Beginner
- ○ Basic (I followed some machine learning courses)
- ○ Advanced (I work on training models myself)
- ○ Pro (I know how to tune the model and apply new methods myself)

18. Which applications of AI do you expect in your clinic within the next 5 years? (multiple options possible)

- ☐ Contouring
- ☐ Planning
- ☐ Machine QA
- ☐ Synthetic CT
- ☐ Decision making
- ☐ None

Optional comment:

19. What is needed for broader adoption of AI in clinic (please rank in order of importance)

- Training medical doctors and technicians
- Training medical physicists
- Employ AI experts
- Introduce patient specific QA tools (i.e. independent check)
- Introduce man-machine interfaces
- Larger databases including multi-center data
- Better AI models

20. Do you think ESTRO should offer a course on Machine Learning/ Deep Learning?

- [ ] No, there are plenty of courses available
- [ ] Yes, for physicists to increase knowledge or get started on building and training your own models
- [ ] Yes, for physicists to aid in commissioning and implementation of models in your clinic
- [ ] Yes, multidisciplinary on how to use models in the clinic and what to expect from it
- [ ] Other suggestions:

21. Do you think ESTRO should provide guidelines on QA and use of Machine Learning/ Deep Learning?

- ( ) No
- ( ) Yes

Other suggestions:
